# About an aggressive bone lesion

**DOI:** 10.1002/ccr3.1197

**Published:** 2018-03-07

**Authors:** Dhia Kaffel, Hela Kchir, Wafa Hamdi, Imen Zouch, Kaouther Maatallah, Mohamed Montacer Kchir

**Affiliations:** ^1^ Institut National Mohammed Kassab d'Orthopedie La Mannouba Tunisia; ^2^ Hopital la Rabta Tunis Tunisia

**Keywords:** Brown tumor, hypercalcemia, hyperparathyroidism, lytic bone lesion

## Abstract

We report here a case of hyperparathyroidism with disseminated brown tumors mimicking malignancy. The important clinical teaching of our case is that hyperparathyroidism can take various aspects. Plasma parathyroid hormone concentration should be measured in all patients with multiple bone lesions.

A 77‐year‐old man, hypertensive, hospitalized for a left hip pain. He also suffers from constipation 2 years ago. The examination shows a painful and limited range of movement of this hip. In biology: hypercalcemia at 3.87 mmol/L (normal range, 2.2–2.6 mmol/L), hypophosphatemia at 0.6 mmol/L (normal range, 0.8–1.5 mmol/L), and increased alkaline phosphatases level of 492 IU/L (normal range, 42–160 IU/L). C‐reactive protein and erythrocyte sedimentation rate were within the normal range. The radiograph of the pelvis showed a lytic lesion of the femoral neck (Fig. 1). The thoraco‐abdomino‐pelvic CT scan finds multiple lytic bone lesions with cortical rupture and soft tissue invasion (ribs, clavicle, ischio‐pubic branch, iliac wing, head, and femoral neck; Figs 2 and 3). In view of this clinical situation, a metastasis, a myeloma, or hyperparathyroidism were considered. The parathyroid hormone (PTH) was increased (94.4 pmol/L (normal range, 1.6–6.9 pmol/L)). The cervical ultrasonography showed a right parathyroid nodule. The diagnosis of hyperparathyroidism with disseminated brown tumors was retained. The outcome was favorable after parathyroidectomy with normalization of postoperative serum calcium and PTH.

**Figure 1:**
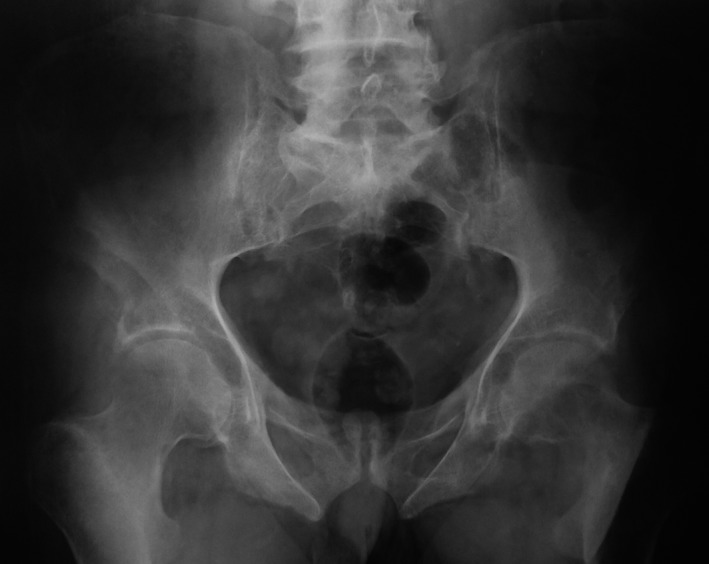
Radiograph of the pelvis: lytic lesion of the left femoral neck.

**Figure 2:**
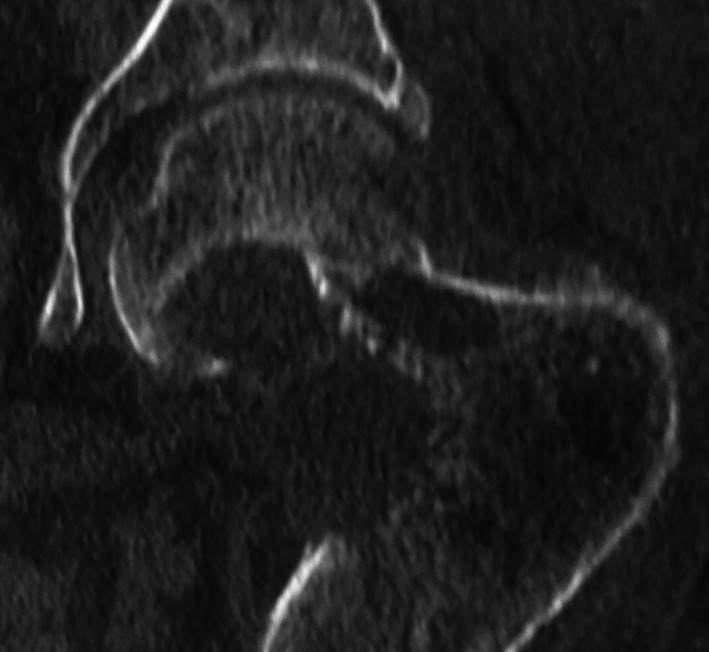
CT scan of the head and femoral neck: lytic bone lesions with cortical rupture and soft tissue invasion.

**Figure 3:**
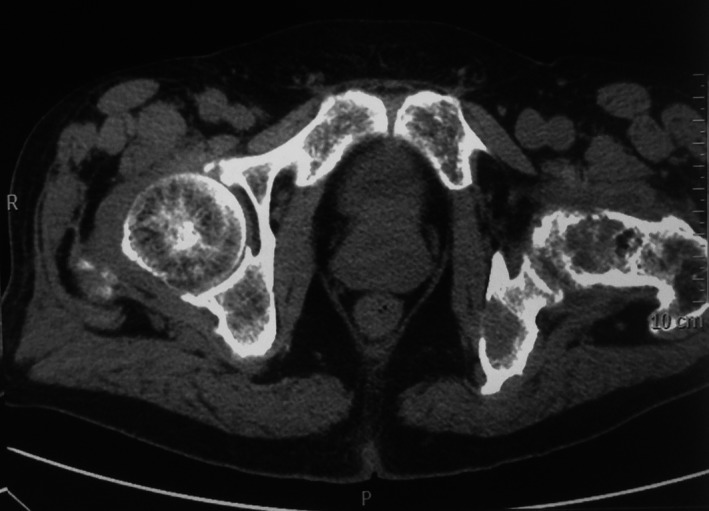
CT scan of the pelvis: multiple lytic bone lesions with cortical rupture and soft tissue invasion.

The important clinical teaching of our case is that hyperparathyroidism can take various aspects. Plasma parathyroid hormone concentration should be measured in all patients with multiple bone lesions 1.

## Conflict of Interest

None declared.

## Authorship

DK: submitted the manuscript, took the pictures with the help of all co‐authors, also wrote the text with the help of all co‐authors. HK: was the first doctor who saw the patient, helped us to make the final diagnosis, also participated in taking pictures and writing the text. WH: helped us to make the final diagnosis, participated in taking pictures and writing the text, also supervised all the work. IZ and KM: helped us to make the final diagnosis, also participated in taking pictures and writing the text. MMK: supervised all the work.
